# Ojeok-san ameliorates visceral and somatic nociception in a mouse model of colitis induced colorectal cancer

**DOI:** 10.1371/journal.pone.0270338

**Published:** 2022-06-23

**Authors:** Patrice Cunningham, Aman Sumal, Emma Patton, Henry Helms, Matthew T. Noneman, Gustavo Martinez-Muñiz, Jackie E. Bader, Ioulia Chatzistamou, Ahmed Aladhami, Christian Unger, Reilly T. Enos, Hyeun Kyoo Shin, Kandy T. Velázquez

**Affiliations:** 1 Department of Pathology, Microbiology, and Immunology, University of South Carolina School of Medicine, Columbia, South Carolina, United States of America; 2 Basic Herbal Medicine Research Group, Korea Institute of Oriental Medicine, Daejeon, Republic of Korea; University of Tennessee Health Science Center, UNITED STATES

## Abstract

Cancer patients can develop visceral, somatic, and neuropathic pain, largely due to the malignancy itself and its treatments. Often cancer patients and survivors turn to the use of complementary and alternative medicine (CAM) to alleviate pain and fatigue. Thus, it is necessary to investigate how CAM therapies work as novel analgesics to treat cancer pain. Ojeok-san (OJS) is an herbal formula consisting of seventeen herbs. This herbal formula has been shown to possess anti-inflammatory, immunoregulatory, and analgesic properties. In this study, we examined the potential beneficial effects and mechanism of action of OJS in a preclinical model of colitis-associated colorectal cancer. Male and female C57BL/6J mice were exposed to the carcinogen, azoxymethane (AOM, 10 mg/kg) and a chemical inflammatory driver, dextran sulfate sodium (DSS1-2%), to promote tumorigenesis in the colorectum. OJS was given orally (500, 1000, and 2000 mg/kg) to determine its influence on disease activity, tumor burden, nociception, sedation, Erk signaling, and behavioral and metabolic outcomes. In addition, *in vitro* studies were performed to assess CT-26 cell viability, dorsal root ganglia (DRG) activation, and bone-marrow-derived macrophage (BMDM) inflammatory response to lipopolysaccharide stimulation after OJS treatment. We found that administration of 2000 mg/kg of OJS was able to mitigate mechanical somatic and visceral nociception via Erk signaling without affecting symptom score and polyp number. Moreover, we discovered that OJS has sedative properties and elicits prolonged total sleeping time in AOM/DSS mice. Our *in vitro* experiments showed that OJS has the capacity to reduce TNFα gene expression in LPS-stimulated BMDM, but no changes were observed in DRG spike number and CT-26 cell proliferation. Taken together, these data suggest that OJS ameliorates nociception in mice and warrants further examination as a potential CAM therapy to promote analgesia.

## Introduction

In the United States there are approximately 1 million colorectal cancer survivors [1,2]. This is the result of an increase in screening and improved cancer treatments [1]. However, although the overall death rate has decreased, there has been an increase in the mortality rate of colorectal cancer patients under 55 years of age [1]. Among the symptoms related to colorectal cancer, pain and fatigue are the most described ailments in patients with advanced cancer [3]. Data from the Cancer Care Outcomes and Surveillance study has revealed that approximately 42% of the colorectal cancer patients suffer from pain and 65% experience fatigue six-months following cancer diagnosis with or without treatment [4]. Pain can also be observed in colorectal cancer survivors (1–10 years post treatment completion) with a prevalence ranging from 7–27% [5–7]. Interestingly, cancer survivors who sleep less than 7 hours at night present more physical pain than those who report sleeping 7 hours or more nightly [8]. Pain relief can be achieved effectively in the majority of patients experiencing minor pain by the use of over-the-counter drugs, such as acetaminophen, ibuprofen, naproxen, and aspirin [9]. However, the feasibility and effectiveness of opioids for the treatment of chronic pain is less clear [9]. For example, in some cases cancer survivors are not able to receive sufficient pain medication to control their pain [10]. Another problem is that long-term opioid therapy is known to increase the risk of overdose and opioid abuse [11]. Therefore, a significant number of colorectal cancer patients (39%) and survivors (78%) have opted to use complementary treatments (e.g., massage, acupuncture, herbal remedies) to improve their pain and quality of life [5,12]. Nevertheless, because complementary medicine has pleiotropic effects throughout the body, little is known with respect to the mechanism by which complementary medicine may alleviate cancer pain.

The medicinal herb, Ojeok-san (OJS) is used in Asian countries to treat pain, gastrointestinal problems, and depression [13]. In fact, OJS is one of the main herbal formula prescribed in Korean medical clinics to treat low back pain [2,14]. Experimental studies have reported analgesic, anti-pyretic, anti-inflammatory, and anti-metastatic properties of OJS with little or no-toxicity [15–23]. OJS has been shown to promote analgesia at 300 and 600 mg/kg, but not at 150 mg/kg of body weight in the pre-clinical model of acetic acid-induced arthritis [24]. Regarding OJS anti-inflammatory properties, studies utilizing models of atherosclerosis and systemic inflammation have shown that OJS treatment is capable of reducing TNF-α, IL-1β, IL-6, and PGE2 in rodents [25,26]. *In vitro* studies utilizing OJS have found an increase in apoptosis and reduction of inflammation via the activation of caspase-3 and a decrease in cytokine and macrophage-derived chemokines, respectively [27,28]. Caspase-3 activation has been associated with both, pro-inflammatory responses and the reduction of inflammation [29–31]. For example, in a neurodegenerative model caspase-3 promoted cell death and microglial activation [31]. In clinical trials, OJS has been given in conjunction with other analgesics (celecoxib and acetaminophen), to investigate safety, tolerability, and pharmacokinetics due to the steady increase of complementary and alternative medicine use in chronic painful conditions [32,33]. Results showed that co-administration of OJS with commonly used analgesics, celecoxib and acetaminophen, is safe [32,33]. While we can infer that part of the analgesic effects of OJS are due to its anti-inflammatory properties, the mechanism by which OJS produces its analgesic effects remains unknown.

The purpose of this study was to investigate the analgesic properties of OJS with respect to colitis-induced colorectal cancer. We first sought to determine the dose at which OJS promotes analgesia without affecting tumorigenesis. Subsequently, to examine referred somatic and visceral nociception, we assessed mechanical threshold and monitored intracolonic pressure changes during colorectal distension. We also studied the possible sedative effects of OJS using the rotarod test and automated behavioral cages. Lastly, a high-throughput *in vitro* platform system was used to determine the mechanism by which OJS promotes analgesia.

## Materials and methods

### Animals

All procedures and experiments involving animals were approved by the Institutional Animal Care and Use Committee at the University of South Carolina. Mice (maximum of 5 per cage) were housed in ventilated cages with wood bedding, nesting material, and *ad libitum* access to food and water. Animals were maintained on a 12:12-h light/dark cycle, 22°C, and 50% humidity.

#### Experiment 1 (Fig 1A)

**Fig 1:**
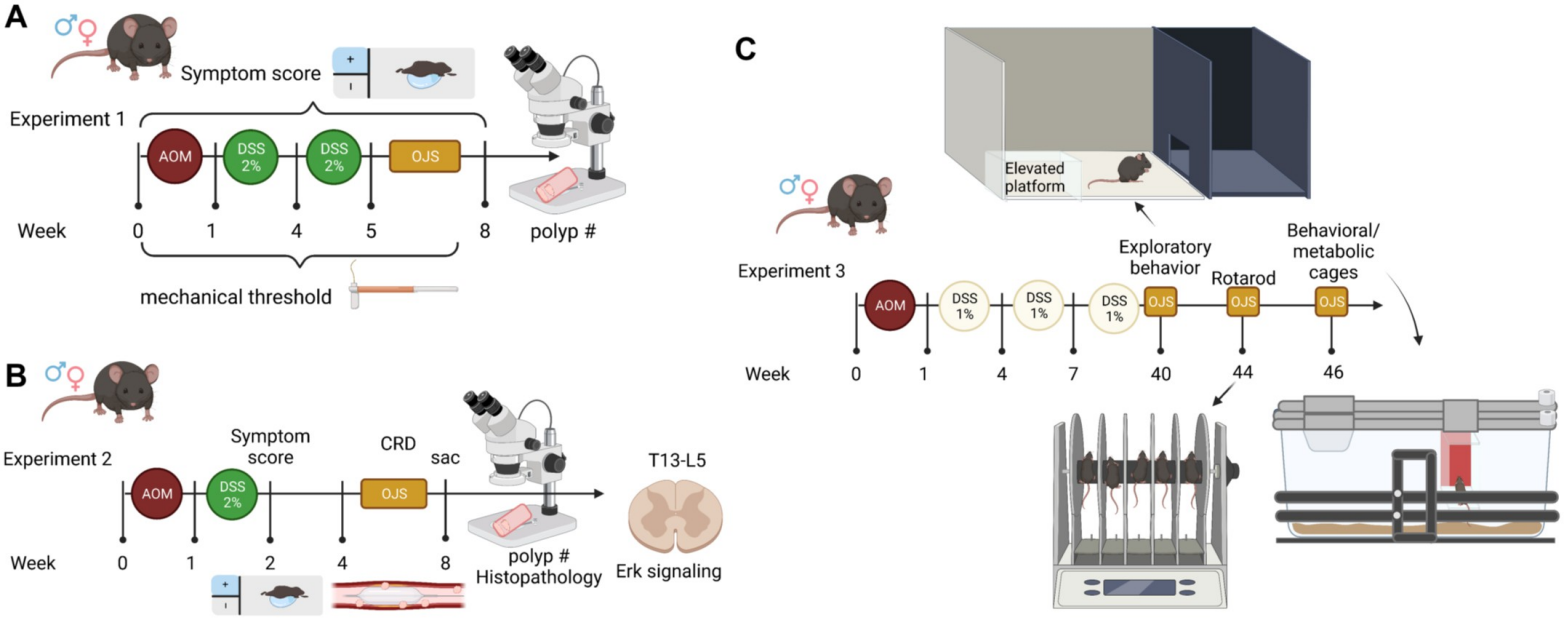
Graphical depiction of the experimental designs. (A) Experiment 1, (B) Experiment 2, and (C) Experiment 3. Created with Biorender.com.

C57BL6/*J* male and female mice (N = 32, n = 4/treatment/sex) were purchased from Jackson Laboratory (Bar Harbor, ME). Mice (10 weeks of age) were separated into four groups following stratified random sampling for sex, body weight, and baseline pain threshold: AOM/DSS-Vehicle (water oral gavage), AOM/DSS-OJS 500 mg/kg (OJS oral gavage at 500 mg/kg of BW), AOM/DSS-OJS 1000 mg/kg (OJS oral gavage at 1000 mg/kg of BW) and AOM/DSS-OJS 2000 mg/kg (OJS oral gavage at 2000 mg/kg). Azoxymethane (AOM, 10 mg/kg, i.p. injection) was administered to AOM/DSS mice (10–11 wks of age) followed by administration of 2% dextran sulfate sodium (MD Biochemicals; approx. 40,000 mol wt) in the drinking water for 7 days at 1 and 4 weeks of treatment. Symptom scores were evaluated every week as described [34]. Referred somatic hyperalgesia (RSH) was performed by one experimenter in a blinded fashion prior the initiation of the experiment and weekly after AOM and DSS treatment. During week five until the end of the study, mice were lightly anesthetized with isoflurane prior to oral gavage of OJS with a plastic feeding tube (22 ga x 55 mm, Instech Laboratories, Plymouth Meeting, PA, catalog # MFG 2018-04-27). Mice were euthanized with isoflurane at 17–18 wks of age (8 wks of treatment) at which time colons were dissected and polyps were counted using a stereoscope.

#### Experiment 2 (Fig 1B)

A total of 40 C57BL/6*J* mice (female, n = 20 and male, n = 20) were obtained from Jackson Laboratories at 8 wks of age. Mice were divided in four groups following stratified random sampling for sex and body weight: Control non-cancer Vehicle (water), Control non-cancer OJS (2000 mg/kg via gavage), AOM/DSS-Vehicle, and AOM/DSS-OJS (2000 mg/kg via gavage). AOM (10 mg/kg of BW) or saline was i.p. injected. DSS (2%) was administered in the drinking water for 7 days as mentioned in Experiment 1. OJS or vehicle was administered a total of five times over the course of the experiment. Symptom score was evaluated as described [34]. Colorectal distension (CRD) was initiated at 14 wks of age (by this time polyps are already formed (unpublished data)). Mice were euthanized with isoflurane and colons were dissected and polyps were counted using a stereoscope. Subsequently, colons were fixed in 4% paraformaldehyde, paraffin embedded, sectioned, and stained with hematoxylin and eosin for histological examination. The thoracolumbar region (T13-L5) of the spinal cord was used to determine Erk signaling. Blood was collected for complete blood count.

#### Experiment 3 (Fig 1C)

Another group of C57BL6/*J* mice (female, n = 20; male, n = 20; 22–23 wks of age from Jackson Labs) were divided into two groups following stratified random sampling for sex and body weight. Mice were injected with AOM or saline as described above and received 1% DSS for 3 cycles. DSS was dissolved in filtered drinking water and administered to mice for seven days at 1, 4, and 8 wks post AOM administration. Exploratory behavior, locomotor activity, and neuromuscular function were assessed using place preference, Promethion behavioral/metabolic cages, and rotarod testing, respectively. OJS was administered via oral gavage (2000 mg/kg of body weight) during exploratory behavior paradigm (1 time gavage with OJS) and rotarod test (1 time gavage with OJS). OJS was administered in the drinking water (15 μg/mL according to water intake-behavioral/metabolic cages) for 10 days.

### Diet and Ojeok-san preparation

All mice were fed a purified AIN-76A diet (BioServ, Foster Lane Flemington, Frechtown) at least two weeks prior to the start of the experiment and throughout the finalization of the studies. OJS was provided by Dr. Hyeun-Kyoo Shin (Director of the Herbal Medicine Formulation Research Group at the Korean Institute of Oriental Medicine). Briefly, seventeen herbal medicines: Atractylodis Rhizoma (*Atracylodes lancea D*.*C*.,7.5 gr), Ephedrae Herba (*Ephedra sinica* Stapf, 3.75), Citri Unshius Pericarpium (*Citrus unshiu* Markovich, 3.75), Magnoliae Cortex (*Magnolia officinalis* Rehd. Et Wils., 3.0), Platycodonis Radix (*Platycodon grandiflorum* A. DC., 3.0) Aurantii Fructus Immaturus (*Citrus* unshiu Markovich, 3.0), Angelicae Gigantis Radix (*Angelica gigas* Nakai, 3.0), Zingiberis Rhizoma (*Zingiber officinale* Rosc., 3.0), Paeoniae Radix (*Paeonia lactiflora* Pall., 3.0), Poria Sclerotium (*Poria cocos* Wolf, 3.0), Angelicae Dahuricae Radix (*Angelica dahurica* Benth. Et Hook. F., 3.0), Cnidii Rhizoma (*Cnidium officinale* Makino, 2.63), Pinelliae Tuber (*Pinellia ternata* Breit., 2.63), Cinnamomi Cortex (*Cinnamomum cassia* Presi, 2.63), Glycyrrhizae Radix et Rhizoma (*Glycyrrhiza uralensis* Fisch. 2.25), Zingiberis Rhizoma Recens (*Zingiber officinale* Rosc., 3.75), Allii Fistulosi Bulbus (*Allium fistulosum* L., 3.75) were mixed, extracted with water, filtered, and freeze-dried to form the OJS powdered formula. The purity and chemical profile of the OJS used in these experiments has been published by Kim *et*. *al*., [35]. The powdered extracts were stored at 4°C. OJS extract was evaluated for pesticide residue and heavy metals. Dichlorodiphenyl-trichloroethane, hexachlorocyclohexene dieldrin, aldrin, endrin, and sulfur dioxide were not detected in the extract. Arsenic (0.21ppm), cadmium (0.04 ppm), and lead (0.76ppm) were detected at concentrations under the maximum tolerable/tolerance level in complete feed according to the National Research Council [36].

### Behavioral and functional tests

#### Referred somatic hyperalgesia (RSH)

RSH was assessed at 0, 1, 2, 3, 4, 5, 6, and 7 weeks of treatment (Experiment 1). Briefly, mice were placed on a raised transparent plastic box (30x20x15 cm) with a bottom made of wire mesh (5 x 5 mm apertures) for 30 min during 3 days of habituation and prior weekly tests. Von Frey filaments (Bioseb, Pinellas Park, FL) 0.008, 0.02, 0.04, 0.07, 0.16, 0.40, and 0.60 grams were applied five times for 5 seconds in ascending order to avoid windup effects of desensitization. A nociceptive response was defined as: sharp withdrawal of the abdomen, licking or scratching the touched area, and/or jumping. The pain threshold was defined as the force in grams at which any von Frey filament elicited three nociceptive responses out of five. Exclusion criteria were defined as a pain threshold higher than 0.4 grams at week three in cancer mice. The same investigator performed all RSH tests (blinded to treatments groups) during the beginning of the light cycle 7-9am. Additionally, the experimenter analyzing the data was blinded to group assignments.

#### Colorectal distension (CRD)

CRD was evaluated as described [37]. Briefly, mice were anesthetized (isoflurane) and a lubricated balloon (2 cm length x 1 cm inflated diameter) was inserted into the colon. Surgical hypoallergenic tape was used to secure the balloon to the base of the tail. Mice were gavaged with vehicle solution (filtered water) or 2000 mg/kg OJS and allowed to recover from anesthesia for approximately 10 min. Subsequently, mice were placed in a plexiglass cage and allowed to move around. Balloon tubing was connected to a barostat (Distender Series II, G&J Electronics) and ascending phasic CRD was initiated (consisted of three 20 sec pulses at 10, 25, 40, 65, and 80 mmHg with five min in between balloon inflation). Each pulse was repeated three consecutive times to minimize variability due to movement-related artifacts. After the CRD paradigm was finalized, mice were again anesthetized to remove the balloon carefully from the colon. All CRD were performed in the morning (7 am–12 pm). The same investigator performed all CRD experiments in which one mouse from each treatment group was assessed per day. A transducer amplifier (Labtrax4, World Precision Instruments, Sarasota, FL) and Data Trax 2 software (World Precision Instruments) were used to collect and analyze intracolonic pressure during the inflation of the balloon, which is related to the abdominal muscle contractions produced by the CRDs [38]. The balloon pressure signal (BalP) was calculated using the area under the curve (for 5 sec period before and after each pulse) of three consecutive pulses. The experimenter analyzing the data for colorectal balloon distension was blinded to the experimental groups.

#### Exploratory behavior

Exploratory behavior was assessed in female and male mice. Mice were habituated for 30 min for three days to a plexiglass box (51 x 26 x 30 cm, custom made by Mike C. Gore, PPN Department Machine Shop at USC). During the behavioral test, mice were placed in the center of the box (25 x 25 x 30 cm) and time to step up to the platform inside the box (25 x 12 x 5 cm) and/or time to enter to a black enclosing inside the box were recorded in seconds. Each mouse performed ten trials with a cut-off of 120 sec if the mouse remained in the center of the box. Percent time mice favored the center, step-up, and black enclosing space were calculated.

#### Behavioral and metabolic phenotyping

Behavioral and metabolic phenotyping were assessed using the Promethion multichannel continuous measurement indirect calorimetry system (Sable System International, Las Vegas, NV, USA). Control (non-cancer) and AOM/DSS mice (male n = 4/group, female n = 4/group) were singly housed for 3 days of acclimation prior to 7 days of data collection. Food intake, water consumption, body mass, total activity, energy expenditure, respiratory quotient, animal ambulatory locomotion, and sleeping patterns during a 12-h light and 12-h dark cycle were collected and analyzed. Body composition was measured using dual-energy X-ray absorptiometry (S5 Fig). Then, the same mice were given OJS in their drinking water and placed again for another 7 days in the Promethion cages. The percentage of each animal’s total time engaged in eating, drinking, inside their habitat, and sleeping were calculated using Expedata and automated analysis scripts (Sable System International, Las Vegas, NV, USA). All data (besides RER and all meters = two-tailed student’s T-test) were analyzed using an ANCOVA with lean mass as a covariate utilizing the MMPC Statistical Analysis Page (https://www.mmpc.org/shared/regression.aspx).

#### Rotarod test

Rotarod test was performed to assess OJS’ sedative properties as described by Martin *et*. *al*., 1993 [39]. This test measure’s neuromuscular function. Therefore, motor impairment can be evaluated in agents that promote muscle relaxation due to sedation. Briefly, during habituation and test days (pre-OJS and post-OJS) each mouse was placed on the rod (Columbus Instruments) followed by a ramping protocol [0–25 rpm (0.02 x g) over a period of 90 s followed by 25 rpm from 90–120 s]. The ramping protocol was repeated 3 times. Each trial was separated by a 2-min rest, and the longest time of the three examinations was recorded for each mouse. Mice were given OJS (2000 mg/kg) or water via oral gavage. Mice were lightly anesthetized with isoflurane during the oral gavage procedure. The rotarod test ramping protocol was examined 10-min after oral gavage to allow the mouse to fully recover from anesthesia.

### Symptom score

The symptom score was evaluated once a week starting at the end of the first cycle of DSS exposure in the drinking water. The symptom score was calculated by adding the recorded scores of weight loss (WL), fecal consistency, and blood in the stool. The following numbers were used to score WL (WL<5% body weight loss = 0, WL 6–10% body weight loss = 1, WL 11–15% body weight loss = 2, WL>16% body weight loss = 4), fecal consistency (pellet = 0, pasty = 2, and liquid = 4), and blood in the stool (assessed with hemoccult kit: negative = 0, positive = 2, and gross bleeding = 4). The higher the symptom score, the worse the animal’s health, which is indicative of cancer progression.

### Cell culture

#### Rat dorsal root ganglia (DRG)

DRG cells were purchased from Cell Applications (San Diego, CA, cat. no. R8820NK-10) and used according to the company’s instructions. Briefly, rat DRGs (80,000) were seeded on cytoview microelectrode array (MEA) plates (Axion Biosystems, Atlanta, GA, cat. no. M384-tMEA-24W) coated with rat neuron coating solution II and plating medium (Cell Applications, San Diego, CA, cat. no. R823P-10 and 029–05). Cells were incubated at 37ºC, 5% CO2 in rat ganglion neuron culture medium (Cell Applications, San Diego, CA, cat. no. R823k-50). The medium was changed every 3–4 days and spontaneous activity of the DRGs (neural spikes) were evaluated approximately two weeks after seeding using a Maestro Edge with the impedance and neuro module (Axion Biosystems, Atlanta, GA, cat. no. MAESTRO384-Z-EDGE). Inflammatory soup (IS: 1 μM bradykinin (cat. no. B3259), 10 μM Prostaglandin E2 (cat. no. P5640), 10 μM Histamine (cat. no. H7125), 10 μM serotonin (cat. no. H9523), and five μM ATP (cat. no. A2384) from Sigma-Aldrich, Atlanta, GA) was used to mimic nociceptive stimuli [40]. An Erk inhibitor (U0126, 10 μM, Sigma-Aldrich, Atlanta, GA, cat. no. 19–147) and OJS (100, 200, 500 μg/mL) were administered to rat ganglion culture medium 15 minutes prior to IS addition to the wells and were kept in the Maestro Edge during which spike number and duration were continuously monitored.

#### CT26 colon carcinoma cells

CT26 cells (American Type Culture Collection-ATCC, Manassas, VA, cat. no. CRL-2630) were used due to their molecular resemblance with human colorectal carcinoma cells [41]. Cells were thawed, cultured, and maintained in complete RPMI-1640 (ATCC, cat. no. 30–2001) with 10% fetal bovine serum (VWR, Randor, PA, cat. no. 97068085) and 1% penicillin/streptomycin (Gibco, Dublin, Ireland, cat. no. 15140422). Electrode plates (96-well cytoview-z, Axion Biosystem, Atlanta, GA, cat. no. Z96-IMP-96B) were used to measure cell impedance (ohms, Ώ) at baseline. Impedance is low when cells are not attached to the plate and impedance is high when cells proliferate. CT26 cells (passage 2) were seeded in 96-well cytoview-z electrode plates at a concentration of 75,000 cells per well. Cell proliferation was tracked for 72 hrs using the Maestro Edge with the impedance module (three biological replicates; each plate contained twelve technical replicates per group). Treatment groups consisted of wells treated with media only, CT26-no treatment control (NTC), CT26-tergazyme (1%, cat. no. 1304–1), and CT26-treated with OJS at 10, 50, 100, 200, and 500 μg/mL. Data was normalized to wells containing media alone using the Axis Z software (Axion Biosystem, Atlanta, GA).

#### Bone marrow derived macrophages (BMDM)

BMDM were isolated from the femur and tibia bones. Bone marrow was flushed from the bones with PBS + 2% FBS. RBC lysis buffer (Sigma, cat. no. R7757) was used to get rid of erythrocytes. Isolated cells were centrifuged and resuspended in DMEM medium containing 1% Pen/Strep, 10% FBS, and 20% L929 conditioned media. Cells (0.5x10^6) were allowed to differentiate for 7 days and then were treated with OJS (50, 100, and 200 μg/mL) for 24 hrs. OJS was removed from the media and LPS (100 ng/mL) was incubated for 24 hrs. Trizol, chloroform, and isopropanol were used to isolate RNA from BMDM cells. Applied biosystem’s high-capacity cDNA kit (Thermo Fisher, cat no. 4368814) was used to convert RNA into cDNA. TNFα and 18s Taq probes (Applied Biosystems) were used to determine gene expression using quantitative real-time PCR.

### Western blot

The spinal cord (T13-L5) was homogenized in Mueller buffer containing a protease inhibitor cocktail (Thermo Scientific, Waltham, MA, cat no. A32963) and total protein was quantified using the Bradford method (Biorad, Hercules, CA, cat no. 5000201). Western blot analysis of 50 μg of spinal cord homogenate was fractionated on 10% SDS-polyacrylamide gels and used to determine Erk activation. Gels were transferred to PVDF membrane at 22 V for 1hr using the Genie electrophoretic transfer system from Idea Scientific. Membranes were stained with Ponceau to verify equal loading and efficiency of the transfer. Membranes were blocked with 5% BSA (total Erk) and 5% milk in Tris-buffered saline and 0.1% Tween 20 (pErk) for 1 hr. Primary and secondary antibodies: Erk (cat. no. 9102; 1:1000), phosphorylated Erk (cat. no. 9106, 1:2000), and anti-rabbit (cat. No. 7074; 1:2000) were purchased from Cell Signaling. Primary antibodies were incubated overnight at 4°C and secondary antibodies were incubated at room temperature. An enhanced chemiluminescent substrate for detection of horseradish peroxidase (Thermo Scientific) and autoradiography film (Santa Cruz, cat no. sc-201697) were used to visualize the antibody-antigen interaction. All samples were run in the same gel (S1 Fig). Films were scanned and blots were quantified using scientific imaging software (ImageJ).

### Blood panel analysis

A complete blood count (CBC) analysis was performed using VetScan HMT (Abaxis, Union City, CA). Briefly, blood was collected from the inferior vena cava at sacrifice using a heparinized needle and was placed on ice in a 1.5 ml tube. Approximately 50 μL of whole blood was used for a three-part differential analysis that included white blood cells (WBC), lymphocytes, monocytes, neutrophils, platelets, red blood cells (RBC), hematocrit, and hemoglobin.

### Statistics

Data were analyzed using Prism 8 software (GraphPad). Data for the symptom score, colon length, and polyp number was analyzed using an unpaired Student’s t-test (AOM/DSS-vehicle versus AOM/DSS-OJS). Analysis of covariance was used for metabolic parameters. A Kruskal-Wallis test was used to analyze the pathological profile. A one-way ANOVA followed by a Tukey’s multiple comparisons test was used to analyze the nociceptive threshold, disease activity, and polyp number (mice experiments) as well as impedance in CT26 cells, and number of spikes produced by DRGs (*in vitro* experiments). A repeated measures Two-Way ANOVA with a Fisher’s LSD post-hoc analysis was used to assess colorectal distension and rotarod outcomes. A mixed-effects model followed by a Sidak post hoc analysis was used to determine differences among cancer and control mice treated with vehicle and OJS in the exploratory behavior test. TNFα gene expression was analyzed using a one-way ANOVA with a Fisher’s LSD post-hoc test. Any statistical data that did not pass the equal variance test (Barlett’s test) were logarithmically transformed and reanalyzed. Data are presented as means ± SE, and level of significance was set at p<0.05.

## Results

### Experiment 1: Effects of OJS on referred somatic hyperalgesia and intestinal tumorigenesis in the AOM-DSS model

#### Owithout altering disease activity and polyp number in the AOM/DSS model

**Fig 2:**
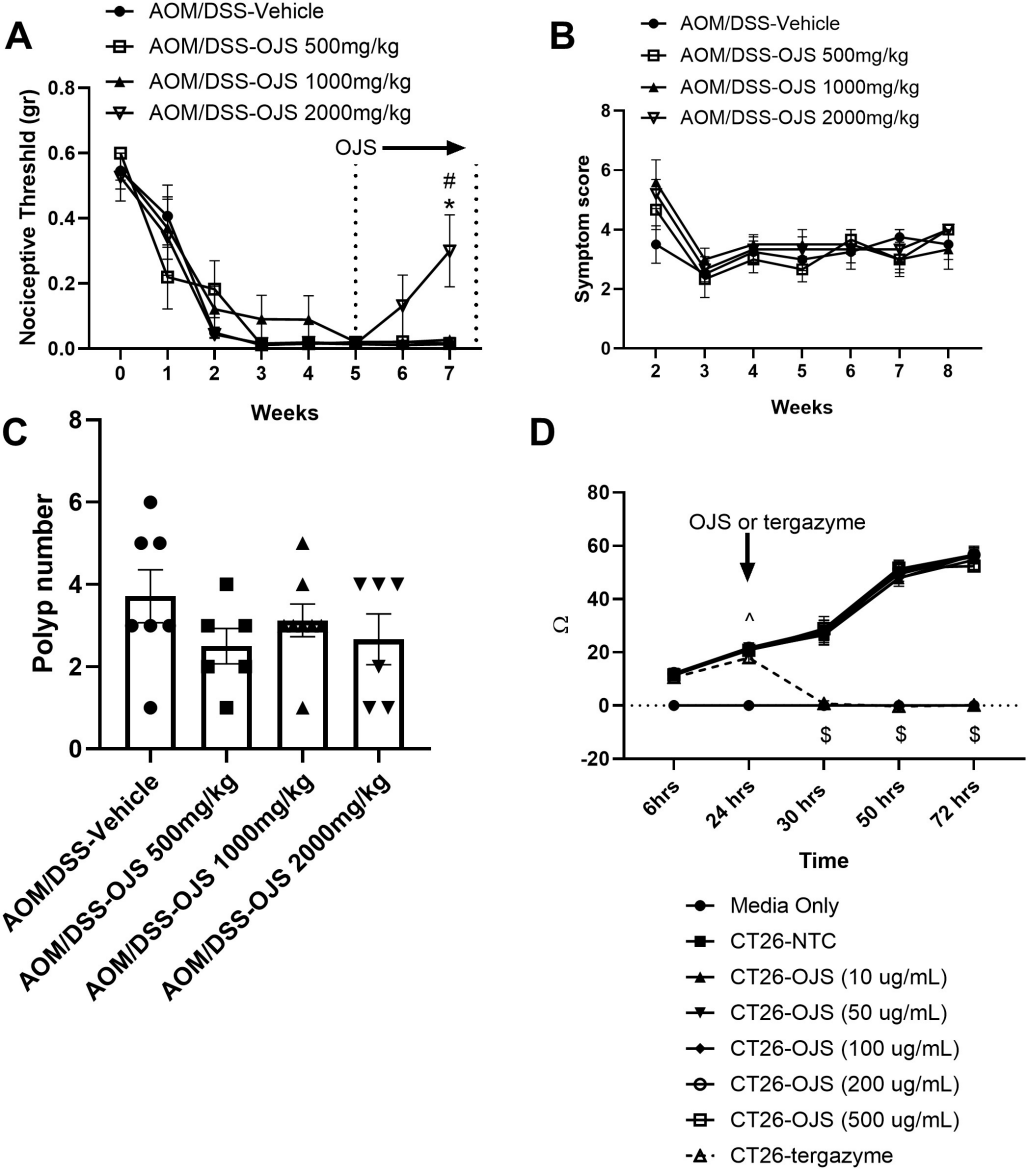
OJS (2000 mg/kg) mitigates referred somatic hyperalgesia in colitis-induced colorectal cancer without impacting tumor burden. (A) Mechanical nociceptive threshold to von Frey filament (0.008, 0.02, 0.04, 0.07, 0.16, 0.40, and 0.60 gr). (B) Disease activity was calculated using score-based symptoms including body weight loss, fecal consistency, and blood in the stool. (C) Polyp count. Number of mice per group as indicated: AOM/DSS-Vehicle, n = 8; AOM/DSS-OJS 500 mg/kg, n = 7; AOM/DSS-OJS 1000 mg/kg, n = 8; and AOM/DSS-OJS 2000 mg/kg, n = 6. * Indicates statistical significance (p<0.05) for AOM/DSS-Vehicle versus AOM/DSS OJS 2000 mg/kg; # indicates statistical significance (p<0.05) for AOM/DSS-OJS 500 mg/kg versus AOM/DSS-OJS 2000 mg/kg from a one-way ANOVA Tukey’s multiple comparisons test. (D) CT-26 cell impedance (proliferation) was evaluated pre- (6 and 24 hrs) and post- (30, 50, and 72 hrs) OJS and Tergazyme (1%, cell lysis) administration. The assay was run in 96-well plate in the presence of OJS at doses ranging from 10–500 ug/mL. Each point represents the mean + SE obtained in three biological replicates; each group consist of twelve technical replicates. ^ Indicates statistical significance (p<0.05) for all groups (CT26) versus media only; $ indicates statistical significance (p<0.05) for CT26-NTC and CT26-OJS (all doses) versus media only and CT26-tergazyme.

To assess whether OJS promotes analgesia in the AOM/DSS colitis-associated colon cancer model, we measured the nociceptive threshold of the abdominal skin of mice using von Frey filaments (Fig 2A). All mice at baseline presented a high nociceptive threshold (approximately 0.6 grams—the highest filament used in this experiment). After the first DSS cycle, the nociceptive threshold started to decrease in all treatment groups. The AOM/DSS-vehicle, AOM/DSS-OJS 500 mg/kg, and AOM/DSS-OJS 1000 mg/kg groups did not show any differences in nociceptive threshold at wks 0–7 of the experimental timeline. However, AOM/DSS mice treated with 2000 mg/kg of OJS for two weeks showed a significant (p<0.05) increase in nociceptive threshold at wk 7 when compared to AOM/DSS-vehicle and AOM/DSS-OJS 500 mg/kg mice.

To address the question whether OJS has the capacity to promote changes in the progression of the disease and/or tumorigenesis, we determined disease activity (symptom score) weekly after the first cycle of DSS, and polyp number at the end of the experiment. In addition, we assessed cell proliferation *in vitro*. No differences in symptom score or polyp number were observed between groups (Fig 2B and 2C).

To examine if OJS modulates cell proliferation, we performed an OJS dose-response i*n vitro* impedance assay in CT26 mouse colon carcinoma cells. Treatment groups were assigned 24 hrs after cell seeding in order to avoid any well-to-well variability with respect to cell resistance/impedance. Impedance in CT26-NTC (non-treatment control) increased over time due to cell proliferation (Fig 2D). The addition of OJS at doses ranging from 10–500 μg/mL elicited similar cell proliferation patterns as CT26-NTC. As expected, tergazyme (1%) treatment promoted a significant (p<0.05) decrease in resistance which is associated with cell lysis. In general, OJS treatment at doses ranging from 10–500 μg/mL did not promote cell death or enhanced cell proliferation in CT26 cells when compared with CT26-NTC at the time points indicated.

### Experiment 2: Effects of OJS on the mechanical responses to colorectal distension in the AOM-DSS model

#### OJS reduces the mechanical response to nociceptive stimuli without altering disease activity and polyp number in the AOM/DSS model

**Fig 3:**
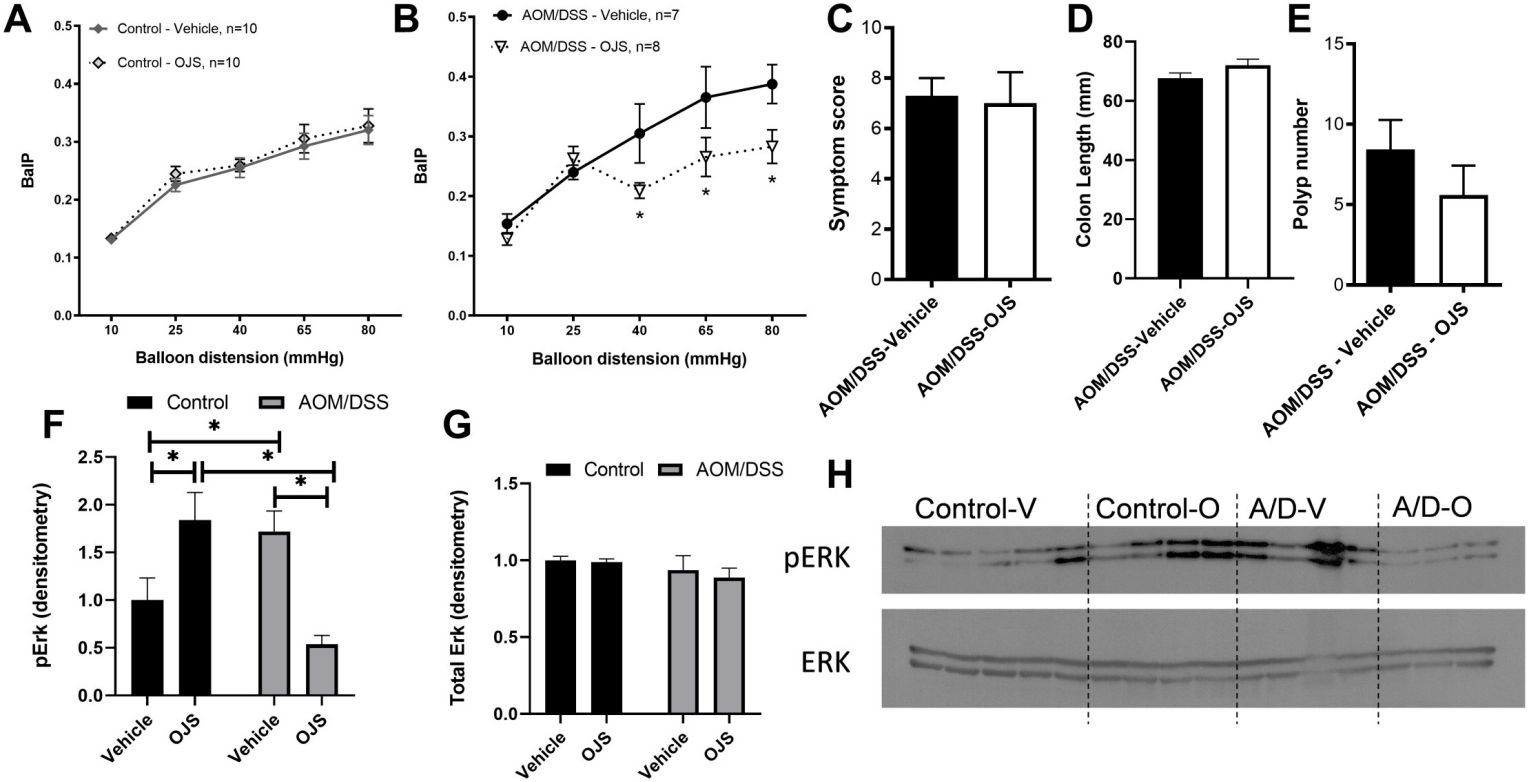
OJS reduces visceral pain-related intracolonic pressure during ascending phasic distensions in AOM/DSS mice. Each colorectal distension (CRD, 10, 25, 40, 65, and 80 mmHg) included a 20 sec duration followed by a 5 min interval between distensions and each distension was repeated 3 times. (A) Control non-cancer mice and (B) AOM/DSS mice were orally gavaged with Vehicle (water) or OJS (2000 mg/kg dissolved in water) 10 min prior to the initiation of CRD. * Indicates statistical significance (p<0.05) from a two-way RM ANOVA Fisher LSD, n = 10 in non-cancer (control) groups, and n = 7–8 in AOM/DSS groups. (C) Symptom score, (D) colon length, and (E) polyp number were assessed in AOM/DSS-Vehicle and AOM/DSS-OJS mice. Spinal cord protein expression of (F) phosphorylated and (G) total Erk and (H) blot images. * Indicates statistical significance (p<0.05) from two-way ANOVA Fisher LSD, n = 4–5 in non-cancer (control) groups, and n = 3–4 in AOM/DSS groups.

We next addressed whether OJS has the capacity to ameliorate elicited visceral nociception in the AOM/DSS colitis-associated colon cancer model. No significant differences were found with respect to intracolonic pressure (BalP) between non-cancer Control-Vehicle, and Control-OJS during CRD at 10, 25, 40, 65, and 80 mmHg (Fig 3A). To compare elicited visceral nociception between non-cancer Control mice and AOM/DSS mice, we calculated the area under the curve (AUC) of intracolonic pressure to CRD (10–80 mmHg). No significant differences were observed between non-cancer Control-Vehicle (8.5±1.4) and AOM/DSS-Vehicle (10.30±2.5) mice (S2A Fig). Additionally, we used histological evaluation of the colon of these mice to determine if histopathological profile correlated to increased elicited visceral nociception. We graphed the highest BalP response to CRD relative to colon pathology. No significant changes in BalP were observed when data was separated by histopathological grade (S2B Fig).

AOM/DSS mice treated with OJS showed a significant (p<0.05) reduction in mechanical responses at 40, 65, and 80 mmHg CRDs when compared to AOM/DSS-Vehicle mice. Control non-cancer Vehicle, Control non-cancer OJS, and AOM/DSS-Vehicle mice displayed a progressive increase in BalP from 25–80 mmHg (Fig 3A and 3B). However, AOM/DSS-OJS mice did not exhibit the same behavior. Consistent with Experiment 1, OJS did not elicit any differences in disease symptoms, colon length, or polyp number in the AOM/DSS model (Fig 3C–3E).

#### OJS reduces Erk1/2 phosphorylation in the thoracolumbar spinal cord in AOM/DSS model

Previous studies have reported activation of extracellular signal-regulated kinases (Erk) 1/2 in the spinal cord of rodents with visceral hyperalgesia [42,43]. Thus, we next tested whether OJS is capable of blocking Erk1/2 phosphorylation in the thoracolumbar region of the spinal cord (T13-L5). As expected, a significant increase (p<0.05) in Erk1/2 phosphorylation was observed between non-cancer Control-Vehicle mice and AOM/DSS-Vehicle mice, but not total Erk (Fig 3G and 3H). Interestingly, we also observed an increase in Erk1/2 phosphorylation in non-cancer Control-OJS mice when compared to non-cancer Control-Vehicle. These results might be because OJS contains cinnamon, which has been shown to activate the TRPA1 receptor [44]. More importantly, administration of OJS was able to decrease Erk1/2 phosphorylation in AOM/DSS-OJS mice when compared to the AOM/DSS-Vehicle and Control-OJS mice.

#### OJS’s impact on blood profile in the AOM/DSS model

**Fig 4:**
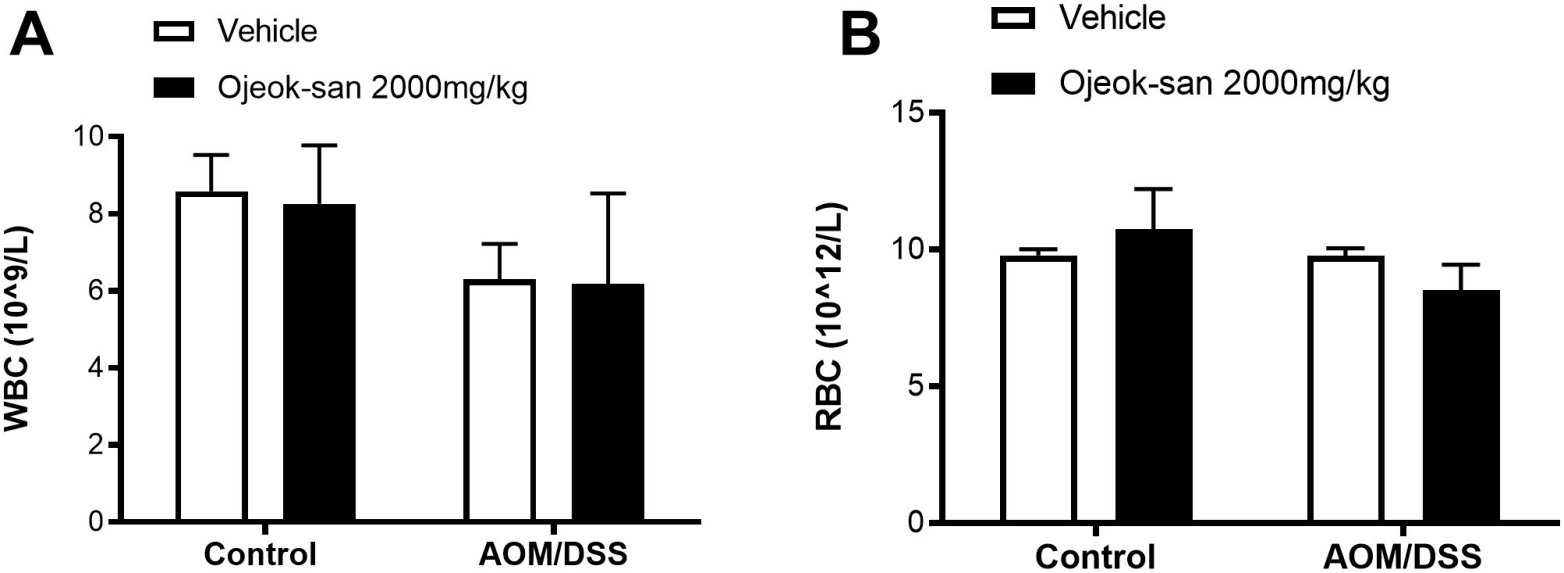
OJS administration did not elicit changes in cell blood counts. (A) White blood cells and (B) Red blood cells were measured using a VetScan HMT. Two-way ANOVA, n = 8–9 in Control non-cancer group and n = 4–7 in AOM/DSS group.

To confirm the safety of OJS, a complete blood count was performed. No differences were detected between groups with respect to WBC and RBC (Fig 4A and 4B).

### Experiment 3: OJS effects on behavioral and metabolic phenotyping in the AOM/DSS model

#### OJS has sedative properties in the AOM/DSS model

**Fig 5:**
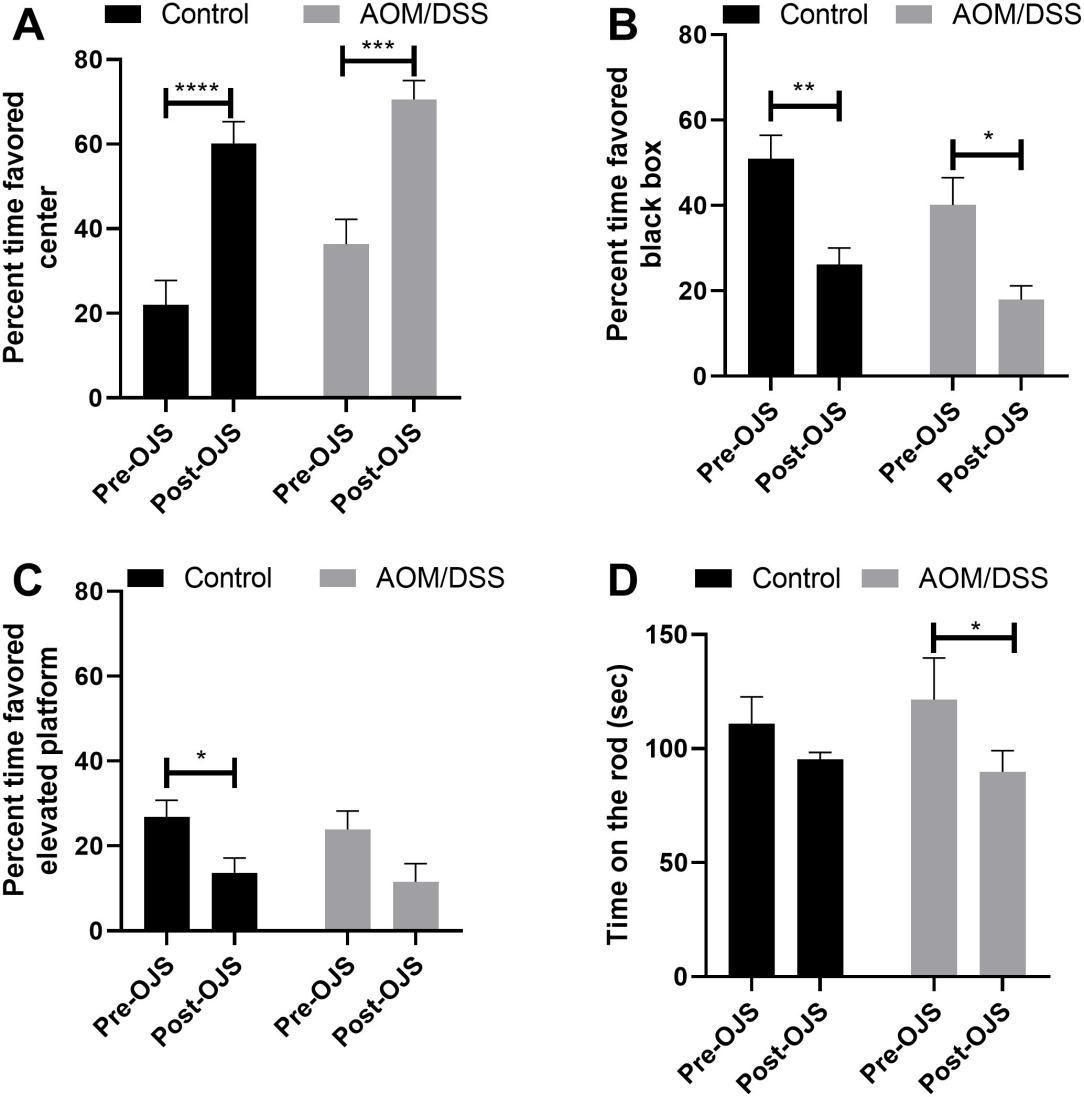
OJS exhibits sedative properties in the AOM/DSS model. Exploratory behavior was assessed using a custom acrylic box with two distinct compartments. Percent time that the mice spent in (A) center, (B) black box, and (C) elevated platform were evaluated at baseline (pre-OJS) and after OJS (post-OJS) administration. n = 17 Control non-cancer mice and n = 13 AOM/DSS mice. * Indicates statistical significance between groups (p<0.05) from Mixed-effects model (REML) Sidak post hoc. (D) Neuromuscular performance in rotarod test from pre-OJS to post-OJS treatment. n = 8 Control non-cancer mice and n = 8 AOM/DSS mice. * Indicates statistical significance between groups (p<0.05) from two-way RM ANOVA Fisher LSD post hoc.

We assessed exploratory behavior utilizing a custom acrylic box with two differential compartments (black box and elevated platform. Percent time that mice favored the black box, platform, or center compartment of the box was calculated from 10 trials (Fig 5A–5C). At baseline (pre-OJS), non-cancer Control and AOM/DSS mice preferred the black box compartment (Control 51% and AOM/DSS 40%) in comparison with center compartment (Control 22%, AOM/DSS 36%) and the platform (Control 27%, AOM/DSS 23%). After oral gavage with OJS (post-OJS) mice switched their preference to the center compartment (non-cancer Control 60% and AOM/DSS 70%) and percent time favoring the black box compartment significantly decreased from baseline (non-cancer Control 26% and AOM/DSS 18%). In addition, non-cancer Control mice also reduced the number of visits to the platform when compared to baseline (Control 14%, AOM/DSS 12%).

To evaluate whether OJS possessed sedative properties we utilized the rotarod test. The rotarod test measures neuromuscular function and it is classically used to examine sedative properties of drugs. For this test, we acclimated the mice to the rotarod and then performed the test 10 min after oral gavage with water (Vehicle) or OJS (Fig 5D). No statistical difference was observed in rotarod latency when water (111 sec) and OJS (95 sec) were given to non-caner Control mice. AOM/DSS mice spent 124 sec on the rod when water was given and significantly reduced the latency time on the rod to 90 sec after OJS administration (p<0.05). From these observations, it is conceivable that OJS may have the potential to act as a sedative.

#### OJS decreases locomotor activity and increases sleep time in the AOM/DSS model, but does not impact indirect calorimetry outcomes

**Fig 6:**
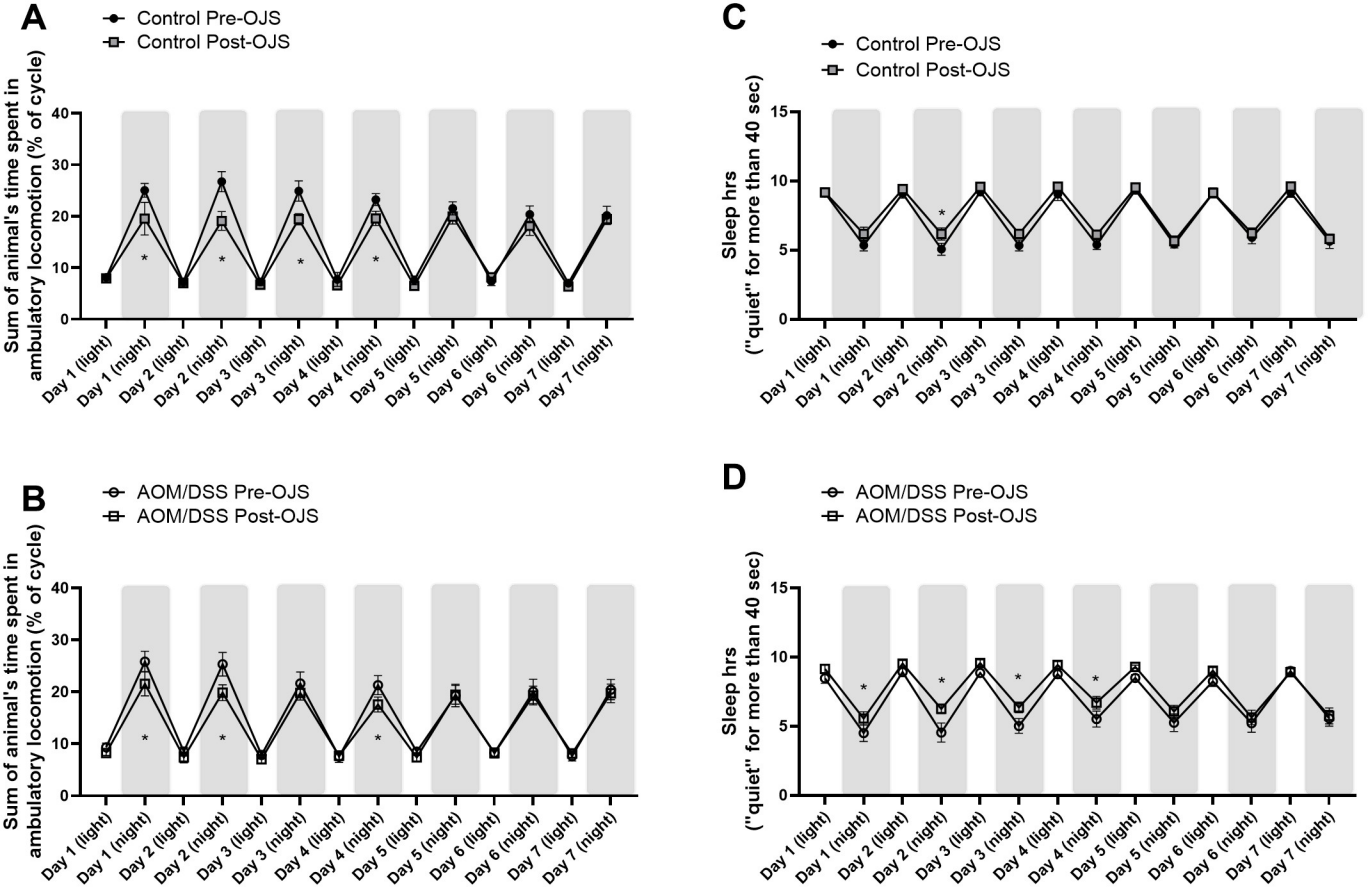
OJS decreases locomotor activity and increases sleep time during the night cycle in the AOM/DSS model. Behavioral phenotyping was achieved by use of the automated Promethion System. Percent time of the cycle that (A) Control non-cancer and (B) AOM/DSS mice spent walking. Total hours that (C) Control non-cancer mice and (D) AOM/DSS mice spent sleeping during the light and night cycle. * Indicates statistical significance between groups (p<0.05) from two-way RM ANOVA Fisher LSD, n = 8/group.

**Fig 7:**
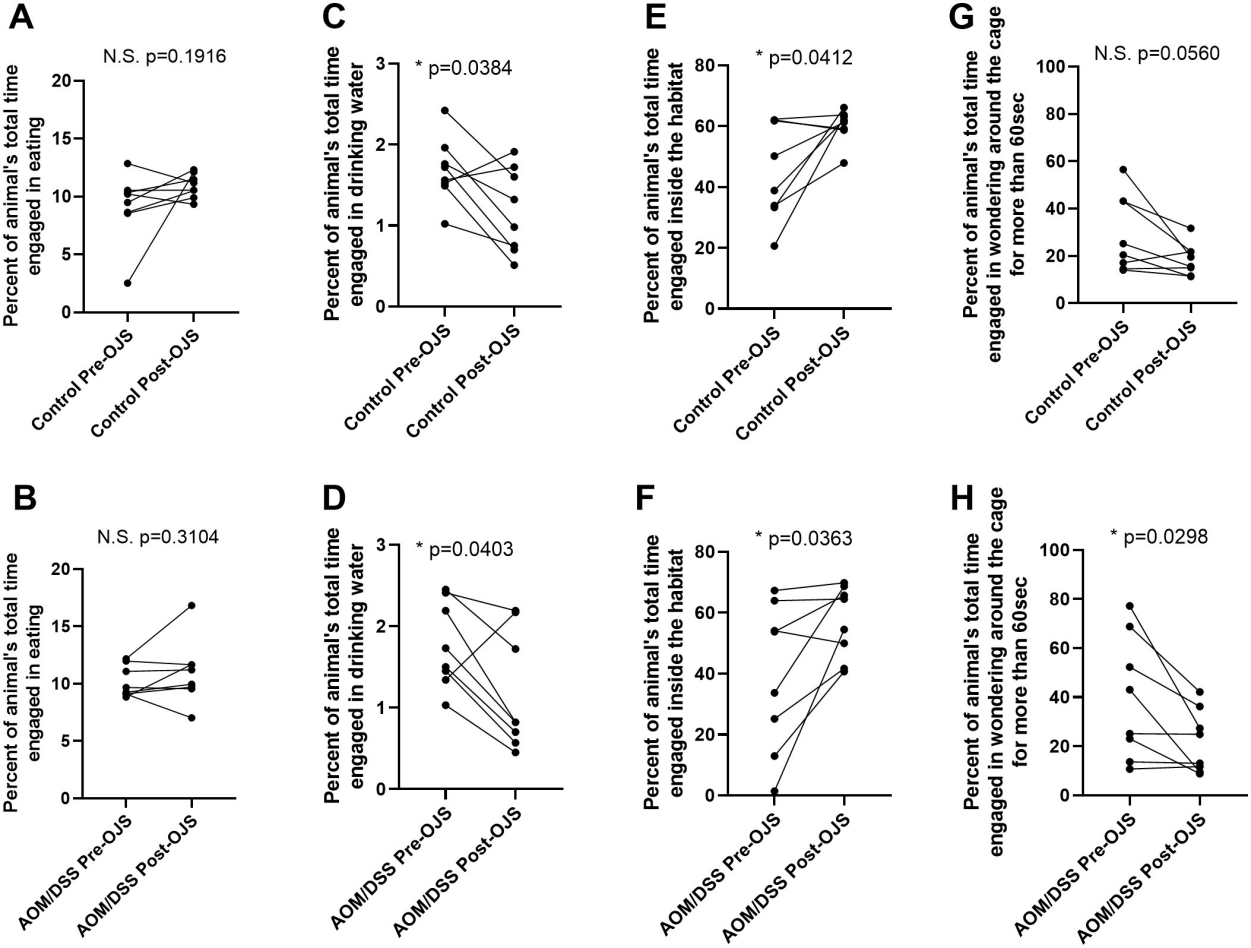
Behavioral time budget pre- and post-OJS administration in the drinking water. Percentage time Control non-cancer mice and AOM/DSS mice spent eating (A, B), drinking water (C, D), inside the habitat (E, F) and long roaming (G, H). * Indicates statistical significance between groups (p<0.05) from paired t-test, n = 8/group.

To better understand the impact of OJS on animal behavior, we utilized the integrated Promethion System consisting of behavioral/metabolic cages. The first week mice were in the behavioral/metabolic cages no treatment was given in the drinking water (pre-OJS). On the second week, mice received OJS in the drinking water (post-OJS). OJS did not impact total energy expenditure, oxygen consumption, or carbon dioxide production (Table 1). However, we observed that OJS treatment significantly decreased ambulatory locomotion during the night cycle (day 1–4) in Control (Fig 6A, P<0.05) and (day 1, 2, and 4) AOM/DSS mice (Fig 6B, P<0.05) when compared with the previous week when all mice did not have OJS in the drinking water (Pre-OJS). When accounting for the time budget allotted to different activities, we found that the AOM/DSS mice significantly (p<0.05) decreased the percentage of time spent moving around the cage (long lounge) during OJS treatment (Post-OJS) (Fig 7H). Control mice showed a reduction in time allocated to locomotor activity, but statistical significance was not observed (Fig 7G, p = 0.0560).

**Table 1:** Metabolic phenotyping of OJS.

	Control pre-OJS	Control Post-OJS	P Value^a^	AOM/DSS Pre-OJS	AOM/DSS Post-OJS	P Value^a^
**Total EE (kcal)**	10.31 (0.153)	9.91 (0.153)	0.088	10.02 (0.249)	9.55 (0.249)	0.198
**O2 Consumption**	1.46 (0.022)	1.41 (0.022)	0.108	1.42 (0.035)	1.136 (0.035)	0.237
**CO2 Production**	1.23 (0.02)	1.21 (0.02)	0.631	1.23 (0.031)	1.19 (0.031)	0.287

EE, energy expenditure.

P-values were calculated using ANCOVA. A total of 8 animals per group (n = 4 female and n = 4 male).

Since sleep has been shown to be a strong predictor of pain [45], we decided to evaluate sleep patterns in these mice. The automated Promethion System defines sleep as time when an animal is not moving (eating, drinking, rooming, locomotion) for more than 40 seconds. Continuous inactivity (≥40 sec) has been previously assessed with video tracking and has shown to be highly correlated with simultaneous electroencephalogram and electromyogram measurements of sleep in mice [46,47]. Non-cancer Control mice slept approximately 9.2 hours during the light cycle and 5.4 hours during the night cycle pre-OJS treatment (Fig 6C). Administration of OJS in the drinking water presented similar sleep patterns in non-cancer Control mice except on the night cycle of Day 2, (p<0.05). On the other hand, AOM/DSS mice slept 8.7 hours during the light cycle and 5.1 hours during the night cycle pre-OJS treatment. AOM/DSS mice treated with OJS slept significantly more (6 hours) during the night cycle (day 1, 2, 3, and 4, p<0.05) when compared to the previous week when they were not given OJS (AOM/DSS Pre-OJS). When accounting for the time budget allotted inside the body mass monitor (or habitat, in-cage enrichment device), AOM/DSS and non-cancer Control mice spent a significantly higher percentage of time inside the body mass monitor when treated with OJS (p<0.05, Fig 7E and 7F). Additionally, we investigated the relationship between intake of OJS in the drinking water and locomotor activity (roam around the cage without interacting with habitat, food, and water monitors for 1–60 sec-short lounge and >60 sec-long lounge) using behavioral transition matrices (S3 and S4 Figs). AOM/DSS mice significantly decreased (p<0.05) the percentage of time they spent on long lounge but not short lounge after OJS intake. No changes with respect to short and long lounge were observed in control mice after OJS consumption in the drinking water.

**Fig 8:**
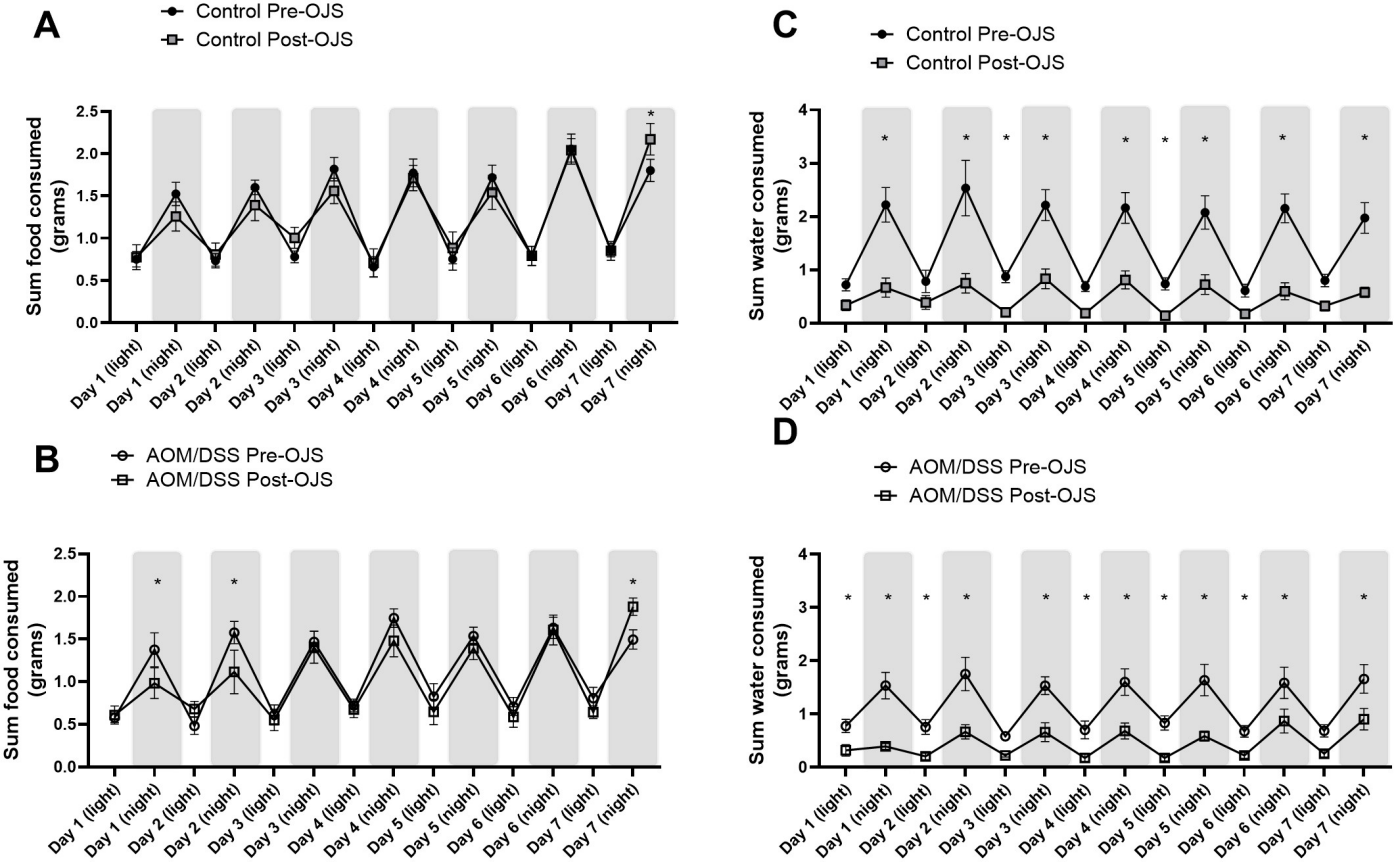
Mice decreased water intake when OJS was added to the water bottle. Food (A, B) and water (C, D) intake in grams during the light and night cycle for control (non-cancer) and AOM/DSS mice. * Indicates statistical significance between groups (p<0.05) from two-way RM ANOVA Fisher LSD, n = 8, control, and n = 8 AOM/DSS.

Non-cancer Control mice ate an average of 1.7 grams during the light cycle and 0.8 grams during the night cycle (Fig 8A). Meanwhile, food intake in AOM/DSS mice was 1.5 grams in the light cycle and 0.7 grams in the dark cycle (Fig 8B). We observed a significant reduction (p<0.05) in the amount of food consumed by AOM/DSS Post-OJS during the night cycle (p<0.05, day 1 and 2). Importantly, we also detected a significant increase (p<0.05) in food intake in non-cancer Control and AOM/DSS mice during the night of day 7. In general, non-cancer Control (1.7 grams) and AOM/DSS (1.4 grams) mice consumed a similar quantity of food and spent comparable amounts of time engaged in eating when OJS was administered in the drinking water (Fig 7A and 7B). Regarding water intake, both non-cancer Control mice and AOM/DSS mice decreased the total time engaged in drinking water and grams of water consumed (during the light and night cycle) when OJS was mixed in the drinking water (Figs 7C, 7D, 8C and 8D).

### OJS does not decrease DRG neuronal firing, but reduces TNFα response to LPS-treated bone marrow macrophages

**Fig 9:**
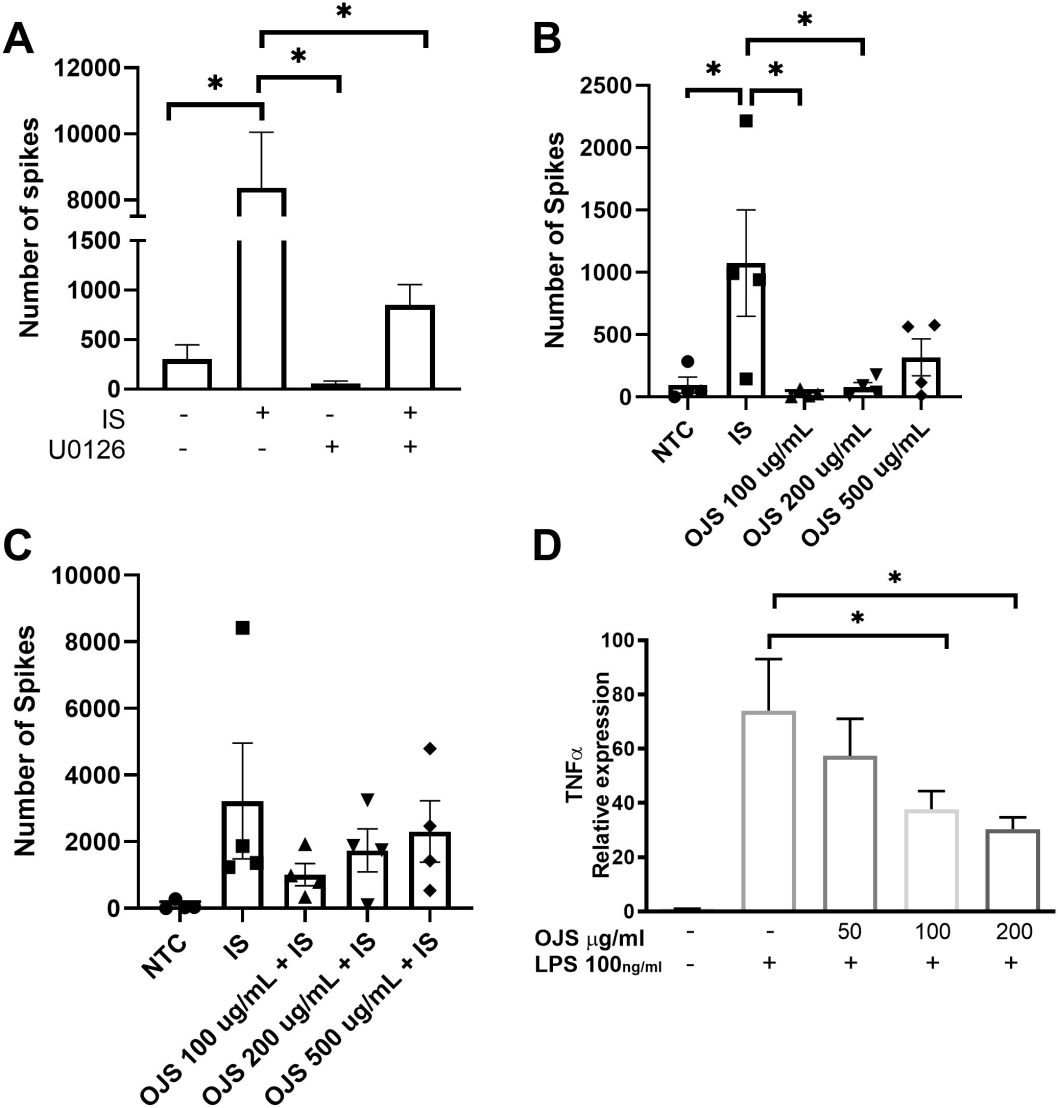
Pre-treatment of OJS did not mitigate DRG activation but decreased inflammatory responses in BMDM. (A) Rat DRG sensory afferents were given inflammatory mediators (IS, inflammatory soup) and U0126 (Erk inhibitor) to determine DRG excitability. (B) The impact of an OJS dose response (100, 200, and 500 μg/mL) on spontaneous DRG firing. (C) Pre-treatment of OJS (100, 200, and 500 μg/mL) 15 min prior to activating DRG afferents with IS. (D) TNFα mRNA expression resulting from LPS-stimulated BMDM. Each bar represents the mean + SE obtained from at least three biological replicates. * Indicates statistical significance between groups (p<0.05) from one-way ANOVA Tukey’s post hoc, n = 3/4 per group.

We used DRG sensory afferent nerves in primary cell culture to study the possible cellular and molecular mechanism by which OJS decreases nociception. Previous studies have reported that inflammatory mediators can evoke firing of the DRG sensory afferent nerves [40,48]. Meanwhile, the Erk inhibitor, U0126, can suppress DRG excitability [40,48]. Given that the Maestro MEA platform can record spontaneous electrical activity in DRG primary cells, we evaluated the impact of inflammatory mediators (IS, inflammatory soup) and Erk inhibition in this system. At baseline DRG sensory afferent nerve presented spontaneous activity with a rate of approximately 200 spikes per min. No significant differences were observed in the number of spikes between the groups at baseline and post administration of the Erk inhibitor (U0126, 10–100μM) (data not shown). As expected, a significant increase (p<0.05) in the number of spikes was observed when inflammatory mediators were given alone (IS) but not in conjunction with the Erk inhibitor (U0126 + IS) (Fig 9A).

To characterize the effects of OJS on spontaneous DRG activity, we performed a dose response experiment with OJS. At baseline, no difference in spike number was observed between groups (data not shown). As previously observed on Fig 9A, IS significantly increased the number of spikes produced by the DRG neurons when compared to NTC (Fig 9B). IS group also presented a significant increase in spike number when compared to OJS 100 μg/mL and OJS 200 μg/mL, but not to OJS 500 μg/mL. OJS at a concentration of 100, 200, and 500 μg/mL did not significantly increase DRG activity when compared to NTC (non- treatment control). Pre-treatment of OJS (100, 200, and 500 μg/mL—15 min prior to IS administration) was not able to decrease neuronal firing caused by IS-stimulation (Fig 9C).

Given that we used an animal model of colitis-induced colorectal cancer and that previous studies have shown that OJS decreases cytokine and macrophage-derived chemokines, we decided to evaluate the impact of OJS on inflammation in macrophages. We used LPS-stimulated bone marrow derived macrophages (BMDM) to assess the anti-inflammatory potential of OJS. Pre-treatment of OJS at 100 and 200 μg/mL was able to reduce TNFα expression, but not at the lower OJS dose of 50 μg/mL (Fig 9D).

## Discussion

Pain is among the most described symptoms in cancer patients and cancer survivors [3–7]. Due to the lack of the feasibility, effectiveness, and numerous adverse effects of pain killers (e.g. non-steroidal anti-inflammatory drugs and opioids) [9], cancer patients and survivors are opting to use complementary treatments to improve their health [5,12]. A greater interest has been observed in utilizing natural compounds for pain management, especially herbal remedies from Traditional Chinese, Korean, and Japanese Medicine that have been used for centuries to decrease pain and inflammation [5,12]. However, distrust in herbal formulas is still prominent due to the lack of scientific evidence supporting the mechanism by which complementary medicine can reduce cancer pain [49]. In this study, we sought to investigate if Ojeok-san (from Korean Medicine, also known as Wu-ji san in Chinese Medicine, and Goshaku-san in Japanese Medicine) decreases pain-like behaviors in mice with colitis-induced colorectal cancer.

To establish OJS’ analgesic properties in a pre-clinical model of colitis associated cancer, we used von Frey filaments and CRD to determine if OJS can decrease somatic and visceral nociception in response to noxious stimuli. These two modalities have been previously used to assess visceral sensitivity caused by colitis and psychosocial stress in rodents [38,50–53]. In addition, colonic hyperalgesia to CRD has been validated in patients with irritable colon syndrome and ulcerative colitis [54,55]. In mice we observed that the nociceptive threshold to the mechanical stimulus decreased to its lowest point by week three of treatment and this pattern continued until the end of the experiment (week 7). Similarly, Eijkelkamp et. al., [50] reported referred hyperalgesia 49 days post-DSS administration. In the past, our group has detected polyps in the AOM/DSS model 2 weeks after the first cycle of DSS (unpublished observations). However, we do not discard that inflammation caused by DSS might be driving somatic nociception instead of the tumor per se. CRD in rats have also shown colonic mechanical sensitivity after exposure to chemical irritants [56–58]. For example, rectal instillation of trinitrobenzene sulphonic acid elicited increased visceromotor response to tonic rectal distension (60 mmHg) as early as 2 weeks post treatment and this pattern continued up to 17 weeks after induction of colitis [56,57]. Increases in colorectal pressures and volumes were also reported in DSS-treated rats as well [59]. In mice, visceral nociception to CRD have been observed after infection with *Trichinella spiralis* [60] and ethanol exposure [61], but not with acetic acid, mustard oil, and DSS alone [62,63]. Likewise, in our mouse model of AOM/DSS colitis-induced colorectal cancer, we did not observe a significant difference in the AUC of intracolonic pressure to CRD between Control non-cancer mice and AOM/DSS mice. Nevertheless, OJS (2000 mg/kg) was able to reduce BalP in AOM/DSS mice. Interestingly, OJS was not able to decrease intracolonic pressure to colorectal distension in Control non-cancer mice at intensities ≥45mmHg which is considered aversive in rodents [52]. This suggests that the mechanism involved in visceral nociception in AOM/DSS mice is different from non-cancer mice. A possibility for this discrepancy may be explained by the fact that cancer promotes peripheral and central plasticity [64].

In many instances, referred somatic hyperalgesia and visceral pain signal through Erk activation [42,43,65]. Thus, we next examined Erk phosphorylation at the level of the spinal cord to further our understanding of the possible mechanism by which OJS promotes analgesia. Of interest was the decrease in Erk phosphorylation from AOM/DSS-OJS mice when compared to AOM/DSS-vehicle mice after colorectal distension. This suggests that OJS might promote analgesia by blunting spinal Erk signaling. Inhibition of spinal Erk activation via intravenous and intrathecal administration of U0126 has previously shown to reduce referred somatic hyperalgesia and visceral nociception to noxious distension [42,65]. In addition, another study reported that U0126 can also reduce repetitive firing in DRG neurons exposed to inflammatory mediators [40]. In this study, we were able to confirm 1) activation of DRGs with inflammatory soup and 2) reduction in the number of spikes when DRGs were pre-treated with U0126. However, OJS pre-treatment at concentrations ranging from 100–500 μg/mL was not able to decrease DRG activation. Therefore, it is possible that OJS might be driving analgesia centrally and not peripherally via the primary afferent nociceptor. Another possibility is that OJS promotes analgesia indirectly by reducing inflammation. Since OJS formula contains seventeen medicinal herbs and some of these herbs have been shown to target transient receptor potential ion channels [66–69], nicotinic acetylcholine receptors [70], adenosine receptor [71], γ-aminobutyric acid receptor A [72], cannabinoid receptors [73], 5HT3 [74] and other pain-related molecules, there is a possibility that OJS is working through multiple mechanisms. In this study we show that OJS was able to decrease TNFα mRNA expression in LPS-stimulated BMDM. Others have reported that OJS can attenuate airway inflammation by inhibiting the recruitment of inflammatory cells [20] and by decreasing cytokine and macrophage-derived chemokines, respectively [26–28]. A limitation of our *in vitro* study is that we only tested OJS 15 min pre-IS and we do not know if changes in duration of OJS treatment can impact neuronal activation. Consequently, more studies need to be done to determine peripheral and central effects of OJS in visceral pain.

Given that OJS has shown anti-tumor activity which can alter nociception and analgesia, we evaluated multiple outcomes associated with disease progression *in vivo* and proliferation *in vitro*. We found no differences in symptom score, polyp number, and colon length between AOM/DSS-vehicle and AOM/DSS-OJS mice. In addition, no changes were observed in the proliferation rate between CT26-NTC cells and CT26 cells treated with OJS at concentrations varying from 10–500 μg/mL. Contrary to these findings, a study conducted in human uterine myomal cells reported that OJS increases the number of dead cells proportionally to OJS concentration [75]. One explanation for the discrepancy between studies is the dose of OJS used. Our highest dose of OJS was 500 μg/mL, meanwhile Jeon et al., 2003 administered OJS at 200, 500, 1000, 2000, and 3000 μg/mL. On the other hand, another study demonstrated that OJS at concentrations from 10–200 μg/mL do not have any effect on cell viability in normal and cancer cells [17]. In general, these data suggest that two weeks of OJS is not sufficient to alter overall tumor burden in mice. As such, future studies using prolonged OJS feeding are necessary to determine possible anti-tumorigenic effects of OJS *in vivo*.

In the present study, we were also interested in examining behavioral and metabolic phenotyping of mice treated with OJS. OJS shifted mice preference from the black box compartment to the center enclosure during exploratory behavior assessment. This result was likely due to OJS’ effects on neuromuscular function as AOM/DSS mice treated with OJS showed a decrease in time spent on the accelerating rod. Additionally, AOM/DSS-OJS mice were less engaged in ambulatory activity and spent more time sleeping. Collectively, these results suggest that OJS possesses sedative (motor impairment) effects. Nevertheless, we cannot abandon the idea that OJS might also be anxiolytic. Interestingly, we did not observe significant differences in motor impairment in Control non-cancer mice. However, Control non-cancer mice treated with OJS reduced locomotor activity during the night cycle. In humans, studies examining the interaction between celecoxib, acetaminophen, and OJS in healthy volunteers reported high tolerability and the most frequent adverse events included feeling hot, sore throat, dry mouth, and headache [32,33]. Since OJS is commonly prescribed in Korean medical clinics for illnesses associated with pain, studies should focus on validating OJS’ side-effects for the development of combinatory treatments in patients that suffer from pain [76].

In general, OJS administration at a concentration of 2000 mg/kg was able to attenuate both referred somatic hyperalgesia and visceral nociception in AOM/DSS mice. Extrapolation of this dose of OJS (2000 mg/kg) that yielded a positive effect on analgesia to a human equivalent dose based on body surface area [77], a commonly utilized method to translate drug dose in animals to humans, would be equivalent to 162 mg/kg. This dose is close to the recommended human dose (196.8 mg/kg/day) in Asian countries [78]. Cytotoxicity and sub-acute toxicity studies in rats demonstrated that this dose does not have adverse effects [17]. We corroborated that no changes in hematological parameters (WBC and RBC) were observed in Control non-cancer and AOM/DSS mice.

## Conclusions

In summary, we establish that OJS has anti-nociceptive effects, which may be mediated, in part, by Erk signaling. Moreover, we show that OJS possesses sedative effects and prolongs the total sleeping time in mice with colitis-induced colorectal cancer. Since OJS has been vastly understudied, further research is necessary to uncover its potential to treat chronic pain. It should be noted that because OJS is a complex formula consisting of 17 herbal medicines, we presume that multiple mechanisms likely contribute to the analgesic properties of OJS.

## Supporting information

S1 Fig Original blot images from Fig 3H. (TIF)

S2 Fig Elicited CRD did not increase intracolonic pressure (BalP) in AOM/DSS mice. (A) Comparison of intracolonic pressure to CRD in control non-cancer mice and AOM/DSS mice. (B) The relationship between BalP response to CRD and colon histopathology.
(TIF)

S3 Fig Behavioral transition matrix for control (non-cancer) mice pre- and post- treated with OJS treatment. (TIF)

S4 Fig Behavioral transition matrix for AOM/DSS mice pre- and post- treated with OJS treatment. (TIF)

S5 Fig Body composition of control and AOM/DSS mice. Lean mass (A), bone mineral density (B), fat mass (C), and (D) percent fat were measured using dual-energy X-ray absorptiometry. Black dots represent male mice and pink dots symbolize female mice. n = 8, control, and n = 8 AOM/DSS.
(TIF)
