# Supplementary figures and images for: Ojeok-san ameliorates visceral and somatic nociception in a mouse model of colitis induced colorectal cancer
Source: PLoS One. 2022 Jun 23;17(6):e0270338. doi: 10.1371/journal.pone.0270338 (PMC9223640; doi:10.1371/journal.pone.0270338)

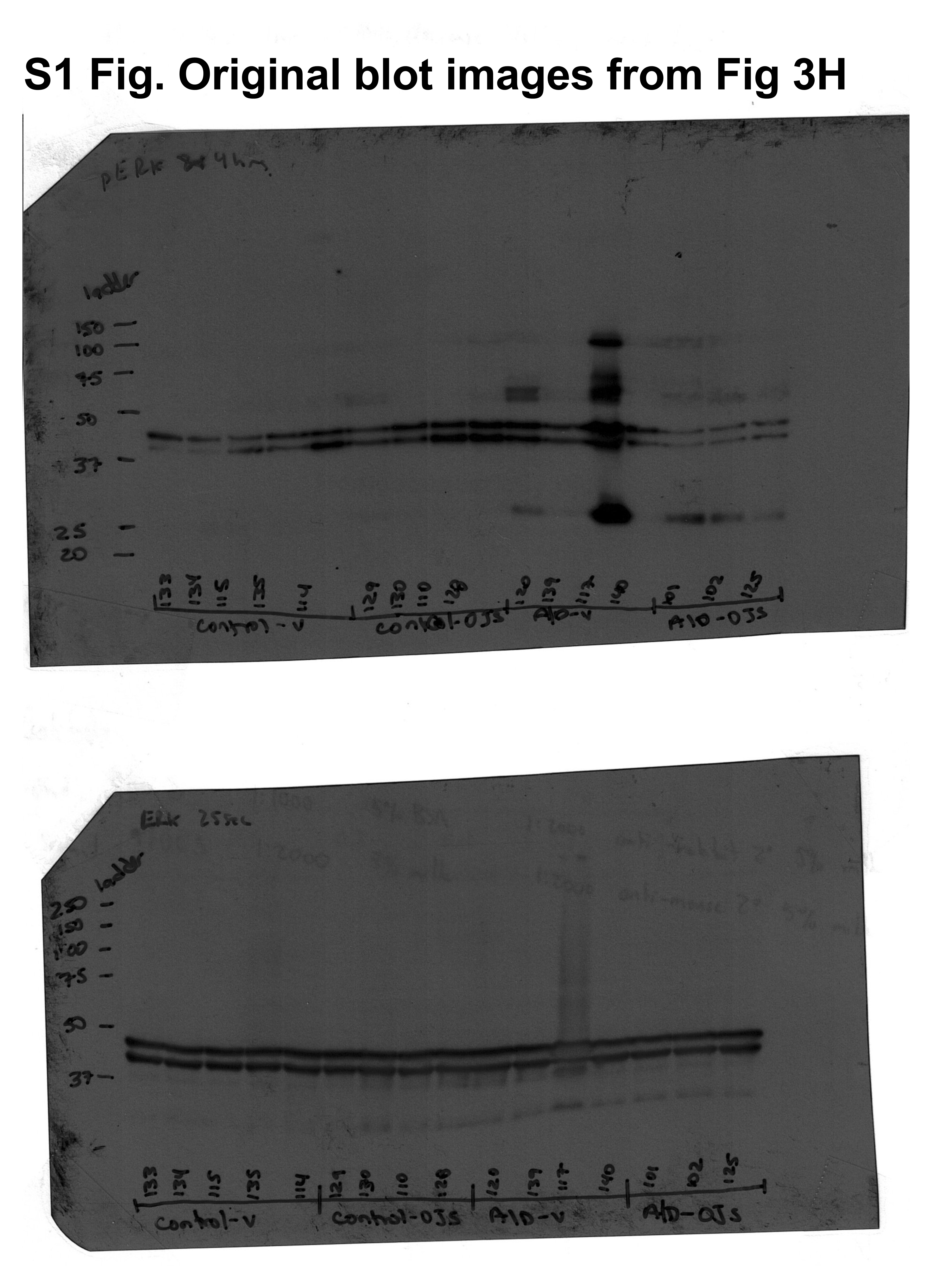

Supplement: S1 Fig — (TIF) [file pone.0270338.s001.tif]

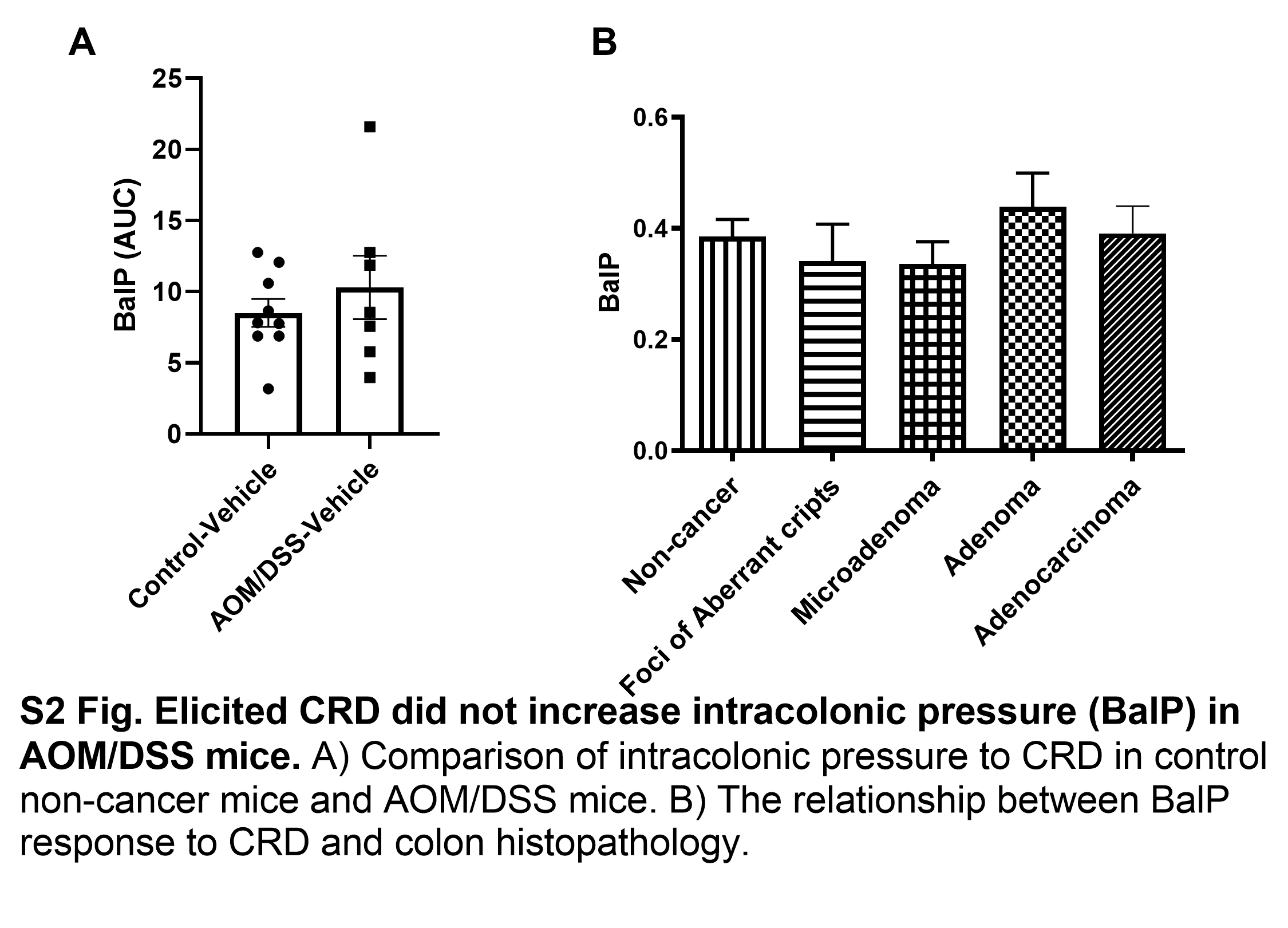

Supplement: S2 Fig — (A) Comparison of intracolonic pressure to CRD in control non-cancer mice and AOM/DSS mice. (B) The relationship between BalP response to CRD and colon histopathology. (TIF) [file pone.0270338.s002.tif]

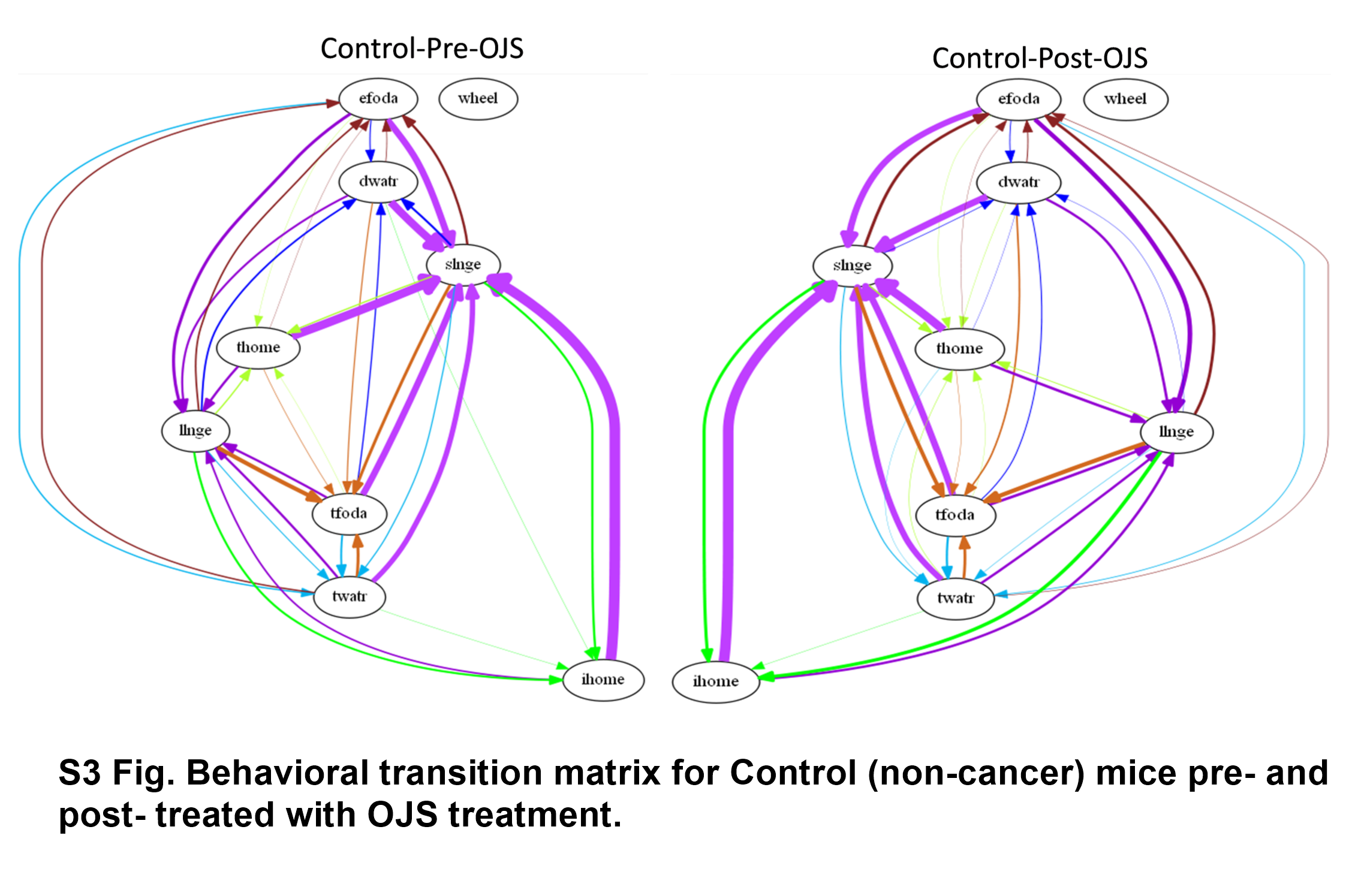

Supplement: S3 Fig — (TIF) [file pone.0270338.s003.tif]

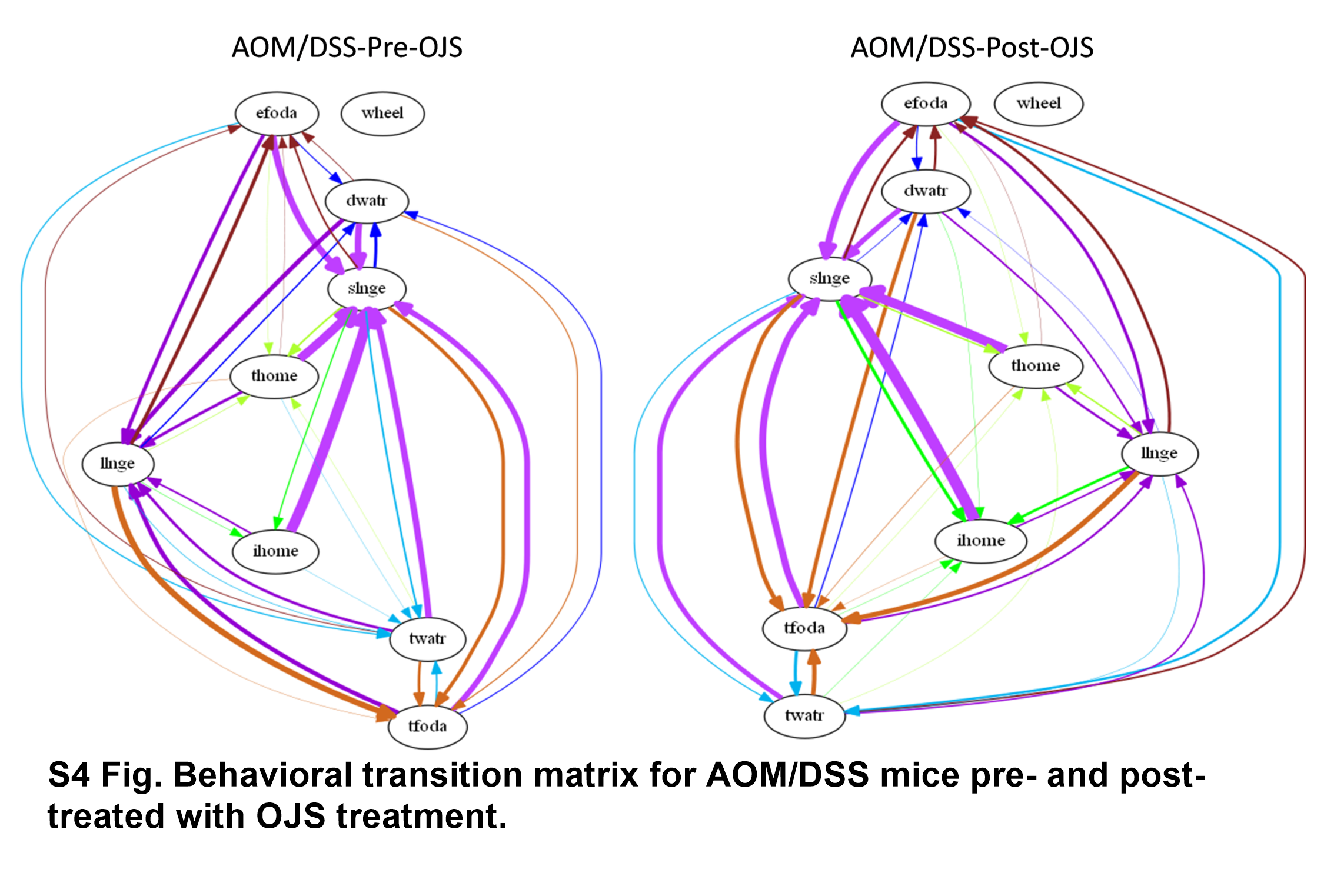

Supplement: S4 Fig — (TIF) [file pone.0270338.s004.tif]

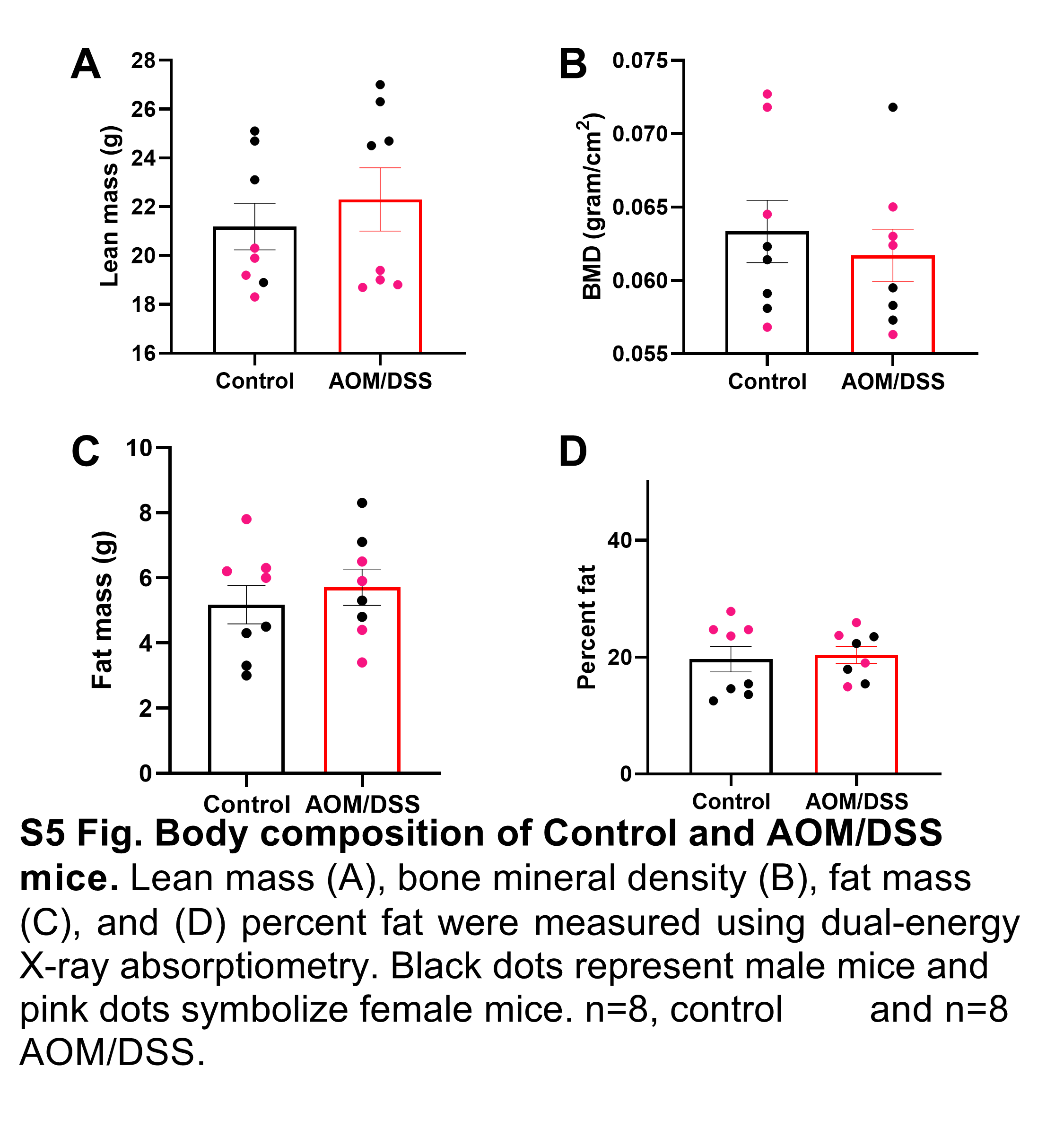

Supplement: S5 Fig — Lean mass (A), bone mineral density (B), fat mass (C), and (D) percent fat were measured using dual-energy X-ray absorptiometry. Black dots represent male mice and pink dots symbolize female mice. n = 8, control, and n = 8 AOM/DSS. (TIF) [file pone.0270338.s005.tif]
